# Predicting who has delayed cerebral ischemia after aneurysmal subarachnoid hemorrhage using machine learning approach: a multicenter, retrospective cohort study

**DOI:** 10.1186/s12883-024-03630-2

**Published:** 2024-05-27

**Authors:** Sihan Ge, Junxin Chen, Wei Wang, Li-bo Zhang, Yue Teng, Cheng Yang, Hao Wang, Yihao Tao, Zhi Chen, Ronghao Li, Yin Niu, Chenghai Zuo, Liang Tan

**Affiliations:** 1 College of Medicine and Biological Information Engineering, Northeastern University, Shenyang, China; 2 School of Software, Dalian University of Technology, Dalian, China; 3 Guangdong-Hong Kong-Macao Joint Laboratory for Emotion Intelligence and Pervasive Computing, Artificial Intelligence Research Institute, Shenzhen MSU-BIT University, Shenzhen, China; 4 Department of Radiology, General Hospital of the Northern Theater of the Chinese People’s Liberation Army, Shenyang, China; 5 Emergency Department, General Hospital of the Northern Theater of the Chinese People’s Liberation Army, Shenyang, China; 6 Department of Neurosurgery, Southwest Hospital, Army Medical University, (Third Military Medical University), Chongqing, China; 7 Department of Neurosurgery, Daping Hospital, Army Medical University, (Third Military Medical University), Chongqing, China; 8 Department of Neurosurgery, the Second Affiliated Hospital, Chongqing Medical University, Chongqing, China; 9 Department of Basic Medicine, Army Medical University, Chongqing, China; 10 Department of Critical Care Medicine, Southwest Hospital, Army Medical University, (Third Military Medical University), Chongqing, China; 11 School of Medical Technology, Beijing Institute of Technology, Beijing, China

**Keywords:** Aneurysmal subarachnoid hemorrhage, Delayed cerebral ischemia, Machine learning, Prediction, Random forest

## Abstract

**Background:**

Early prediction of delayed cerebral ischemia (DCI) is critical to improving the prognosis of aneurysmal subarachnoid hemorrhage (aSAH). Machine learning (ML) algorithms can learn from intricate information unbiasedly and facilitate the early identification of clinical outcomes. This study aimed to construct and compare the ability of different ML models to predict DCI after aSAH. Then, we identified and analyzed the essential risk of DCI occurrence by preoperative clinical scores and postoperative laboratory test results.

**Methods:**

This was a multicenter, retrospective cohort study. A total of 1039 post-operation patients with aSAH were finally included from three hospitals in China. The training group contained 919 patients, and the test group comprised 120 patients. We used five popular machine-learning algorithms to construct the models. The area under the receiver operating characteristic curve (AUC), accuracy, sensitivity, specificity, precision, and f1 score were used to evaluate and compare the five models. Finally, we performed a Shapley Additive exPlanations analysis for the model with the best performance and significance analysis for each feature.

**Results:**

A total of 239 patients with aSAH (23.003%) developed DCI after the operation. Our results showed that in the test cohort, Random Forest (RF) had an AUC of 0.79, which was better than other models. The five most important features for predicting DCI in the RF model were the admitted modified Rankin Scale, D-Dimer, intracranial parenchymal hematoma, neutrophil/lymphocyte ratio, and Fisher score. Interestingly, clamping or embolization for the aneurysm treatment was the fourth button-down risk factor in the ML model.

**Conclusions:**

In this multicenter study, we compared five ML methods, among which RF performed the best in DCI prediction. In addition, the essential risks were identified to help clinicians monitor the patients at high risk for DCI more precisely and facilitate timely intervention.

**Supplementary Information:**

The online version contains supplementary material available at 10.1186/s12883-024-03630-2.

## Background

Delayed cerebral ischemia (DCI) is a common complication of Aneurysmal subarachnoid hemorrhage (aSAH) with a prevalence of up to 30%, sharply deteriorating patient outcomes [1]. Early prediction of patients at risk of developing DCI is critical for maximizing their chances of recovery and reducing the likelihood of permanent brain damage. The pathogenesis of DCI has not been clarified and involves various factors, such as larger and smaller vessel vasospasm, cortical spreading ischemia, microvascular dysfunction, and thrombosis [2]. Therefore, timely recognition and treatment of DCI by preoperative clinical score and postoperative laboratory test is essential to improve the prognosis of patients with SAH [3]. 

Several studies have found that subarachnoid hematoma volume and clinical severity at admission are predictors associated with the development of DCI [4, 5]. And postoperative white blood cell (WBC), neutrophil count, platelets, and erythrocytes correlate with DCI, which could be mild or significant [6–8]. However, these factors were not analyzed as a whole. Some elements may be weakly correlated with the DCI when examined individually. Still, hidden features may improve prediction when all risk factors are diagnosed. Machine Learning (ML) can learn from intricate information and identify concealed characteristics that can enhance forecasts [4, 9]. ML can learn unbiasedly by analyzing numerous variables and samples to generate conclusions [10]. We hypothesized that ML models would be able to learn associations of previously studied correlates to make accurate predictions of DCI.

We used five popular ML algorithms to achieve the following objectives in diagnosis and prediction, namely Random Forest (RF), eXtreme Gradient Boosting (XGBoost), Support Vector Machines (SVM), Gradient Boosting Decision Tree (GBDT), and Decision Tree (DT) [11]. First, we constructed and validated several ML models based on clinical features obtained from clinical and laboratory data at admission and post-operation. Second, we compared the predictive performance of those ML models. Finally, the essential features to predict the occurrence of DCI following aSAH were identified and analyzed based on the most optimized model to help the physicians intervene clinically on time. By utilizing machine learning techniques and conducting a comprehensive analysis of clinical and laboratory data, the identification of fundamental risk factors will assist clinicians in monitoring high-risk patients with DCI more accurately and facilitate timely intervention.

### What is new?

In the multicenter, retrospective cohort study, 1039 post-operation patients with aneurysmal subarachnoid hemorrhage were finally included and analyzed by five machine-learning algorithms. For DCI prediction, RF performed best in the test cohort. The admitted modified Rankin Scale, D-Dimer, intracranial parenchymal hematoma, neutrophil/lymphocyte ratio, and Fisher score are the most important features for predicting delayed cerebral ischemia in the RF model.

### What are the clinical implications?

With our findings, RF had been identified as a superior ML algorithm to predicate DCI following aSAH. The essential risks were identified to help clinicians monitor the patients at high risk for DCI more precisely and to facilitate timely intervention.

## Methods

### Study design and base population

We conducted a retrospective analysis of a cohort of all patients with aSAH admitted between 2010 and 2021 to Southwest Hospital and Daping Hospital, affiliated with Army Medicine University and the Second Affiliated Hospital of Chongqing Medical University, Chongqing, China. We defined our base population as any adult patient (age ≥ 18 years) who was not deceased, was diagnostically identified with aSAH, was admitted to the hospital within 72 h from onset, and underwent an operation for aSAH.

Patients with aSAH with the following conditions were excluded: (1) cerebral infarction on admission, (2) cerebrovascular malformation or hypertensive cerebral hemorrhage, (3) aneurysm-related operation at another hospital before admission, (4) incomplete medical record data, (5) patients who died within three days of admission.

The protocols for vasospasm surveillance were consistent across the participating hospitals, including hourly neurological examinations and daily transcranial Doppler (TCD) ultrasound monitoring and CT angiography.

In accordance with the Helsinki Declaration, the Ethics Committee of the First Affiliated Hospital of Army Medicine University approved the protocol of this study and granted a waiver of informed consent (Approval No: (B) KY2023040).

### Sample size

**Fig. 1:**
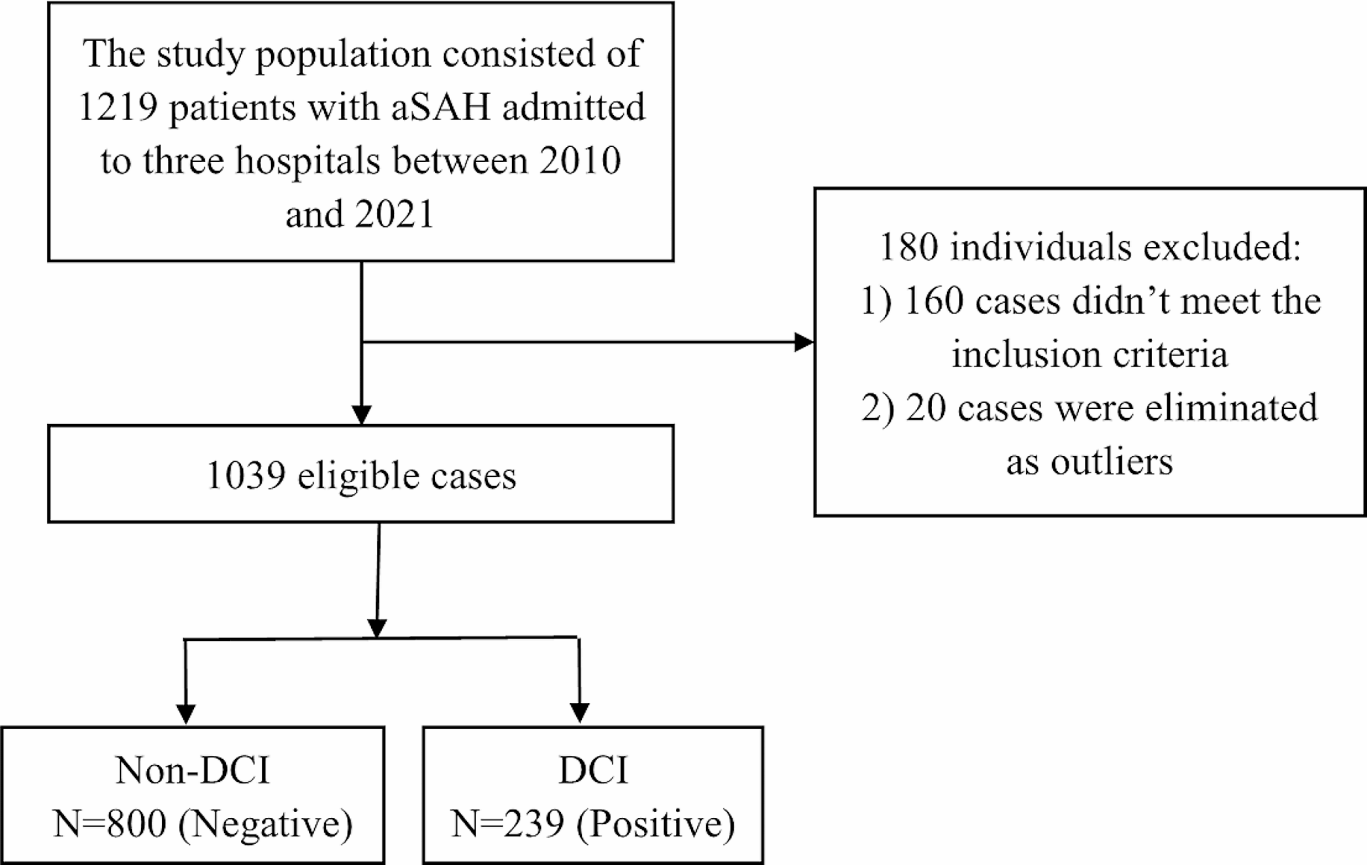
Study flow chart

As a result, a total of 1039 cases were included for analysis. Among the 1039 patients included, 239 (23.003%) developed DCI (Fig. 1). All patients with DCI developed after the operation for aSAH.

### Clinical data

A total of 24 variables were included in this study, including the following admission data: age, sex, blood type, history of smoking, history of drinking, past medical history, history of hypertension, history of diabetes, history of atrial fibrillation, clinical treatment information, admission imaging data that including intracranial parenchymal hematoma and ventricular hemorrhage, clinical condition on admission and clinical routine examination data. The clinical condition on admission was recorded using the Hunt and Hess grade (HH), the World Federation of Neurosurgical Societies (WFNS) scale, the modified Rankin Scale (mRS) after symptom onset, the Glasgow Coma Scale (GCS), and the Fisher score. All clinical routine examination data were collected at the first blood test post operation for aSAH, including D-Dimer, WBC, neutrophil count, lymphocyte count, platelet count, prothrombin time (PT) and activated partial thromboplastin time (APTT). The aneurysm treatment modality included surgical clipping or endovascular coiling for aneurysm repair. The Fisher grading scale criteria are as follows: Grade 1: No blood detected on CT scan; Grade 2: Thin layer of SAH less than 1 mm thick; Grade 3: Localized clot and/or thick SAH more than 1 mm thick; Grade 4: Intracerebral or intraventricular hemorrhage, with or without SAH [12]. 

The appearance of focal neurological deficits (such as hemiplegia, aphasia, apraxia, hemianopia, or neglect) or a decrease of at least 2 points in the Glasgow Coma Scale (in total score or any individual component) lasting for at least one hour, not immediately evident after arterial occlusion, and confirmed through clinical assessment, cranial CT or MRI scans, and appropriate laboratory tests to be unrelated to other causes, defines DCI in present study.

Previous studies have shown that neutrophil/lymphocyte ratio (NLR) [13] and platelet/neutrophil ratio (PNR) are associated with the development of DCI. Therefore, two variables, NLR and PNR, were added to the included variables. To prevent covariance between variables, neutrophil, lymphocyte, and platelet counts were excluded.

### Machine learning methodology

The detection minimum spanning tree method identified and removed outliers from the included patients. To demonstrate the most accurate results, we constructed five different ML models with various ML algorithms using data from two centers: (1) RF, (2) XGBoost, (3) SVM, (4) GBDT, and (5) DT. Each model aimed to identify the patients most likely to have DCI.

### RF

RF is an integrated learning-based algorithm that builds predictive models by constructing multiple decision trees to classify objects sequentially.

#### XGBoost

XGBoost, an integrated learning algorithm based on gradient boosting trees, is an optimized distributed gradient enhancement library designed for efficiency, flexibility, and portability. It implements ML algorithms under the Gradient Boosting framework.

### SVM

SVM is a classical supervised learning algorithm that builds predictive models by constructing optimal hyperplanes or nonlinear decision bounds to classify objects.

### GBDT

GBDT is an iterative decision tree algorithm, which is a gradient-boosting decision tree. It constructs a weak set of learners (trees) and accumulates the results of multiple decision trees as the final prediction output. The algorithm effectively combines decision trees with integrated ideas and has strong generalization capability.

### DT

DT is a tree structure in which each internal node represents a test on an attribute, each branch represents a test output, and each leaf node represents a category.

To develop the ML model, patients were randomly stratified and sampled to obtain the training set and the test set. 20 DCI patients and 20 non-DCI patients were randomly selected from the data of three centers, respectively. As a result, 120 patients were used as the test set, and the rest were used as the training set. The training set was used to develop the model, and the performance of the developed ML model was evaluated on the test set.

To balance the data set, the training set was oversampled. The random oversampling method was used by randomly sampling the minority classes of samples so that the ratio of the two classes was 1:1.

This study used five-fold cross-validation to optimize the model parameters. Five-fold cross-validation, with the parameters determined, divided the data five times and trained five models. These five models were identical except for the different data set divisions, so the five models’ evaluation metrics were obtained. Calculating the variance of the evaluation metrics of the five models and the slight variance indicated that the model had better generalization and was more stable. It also reduced the overfitting of the models to a certain extent.

The model hyperparameters were tuned using GridSearchCV (scikit-learn) to optimize the area under the receiver operating characteristic curve. The grid search algorithm was a method to maximize model performance by traversing a given set of parameter combinations to find the optimal hyperparameter combination.

### Statistical methodology

ML models were developed using the scikit-learn library provided in the Python programming language (v3.9). The performance metrics for this study were sensitivity, specificity, precision, recall, f1-score, and the area under the receiver operating characteristic curve (AUC). The clinical routine examination data were treated as continuous variables, and other variables were modeled as binary or multivariate features.

Shapley additive explanation (SHAP) analysis is adopted to interpret model predictions’ reliability and importance. It provides an intuitive way to help understand the model’s predictive sensitivity to specific features. SHAP values can be used to visualize the contribution of samples and features in the model predictions and thus better interpret the prediction results. To improve the interpretability of the model with the best performance among the five models, we calculated Shapley values for all features to quantify each feature’s importance to the model’s classification.

## Results

### Demographics and data split approaches

Overall, 1039 patients were included in this study, as shown in Fig. 1. The baseline characteristics of all these patients are shown in Table 1. The number of patients with DCI was 179 (19.478%) for the training cohort and 60(50%) for the test cohort. Among them, 663(63.811%) were female, and the two groups accounted for 596 (64.853%) of the training cohort and 67(55.833%) of the test cohort, respectively. The median age of the training cohort was 57(± 8)years, and that of the test cohort was 57(± 8) years. According to the distribution of samples, homogeneity of variance, and sample size, the analysis method is intelligently selected to study whether the differences of various indicators in different groups are statistically significant. When the P-value is < 0.05, the inter-group differences are considered statistically significant. In terms of other features, there were more patients with ventricular hemorrhage in the training cohort than in the test cohort (*p* < 0.001), and the D-Dimer, NLR, HH, Fisher, GCS and mRS were also different among the training and test cohorts.

**Table 1:** Patients’ baseline characteristics in training and test cohorts

Characteristics		Total (*n* = 1039)	Test cohort (*n* = 120)				Training cohort (*n* = 919)		P-value
**Demographics**									
age		57[51, 67]	57 [49, 65]				57 [51, 67]		0.236
sex (Famale)		663(63.811)	67(55.833)				596(64.853)		0.053
Blood type									0.89
A		336(32.339)	42(35.000)				294(31.991)		
B		215(20.693)	24(20.000)				191(20.783)		
AB		90(8.662)	12(10.000)				78(8.487)		
O		333(32.050)	36(30.000)				297(32.318)		
Unknown		65(6.256)	6(5.000)				59(6.420)		
**Medical history**									
smoking		288(27.719)	35(29.167)				253(27.530)		0.706
drinking		276(26.564)	33(27.500)				243(26.442)		0.805
History of illness		581(55.919)	59(49.167)				522(56.801)		0.113
diabetes		49(4.716)	8(6.667)				41(4.461)		0.284
hypertension		521(50.144)	55(45.833)				466(50.707)		0.315
Atrial fibrillation		9(0.866)	2(1.667)				7(0.762)		0.314
Intracranial parenchymal hematoma		173(16.651)	29(24.167)				144(15.669)		0.019
Ventricular hemorrhage		479(46.102)	38(31.667)				441(47.987)		< 0.001
aneurysm treatment modality									0.085
Clipping		644(61.983)	83(69.167)				561(61.045)		
Coiling		395(38.017)	37(30.833)				358(38.955)		
**Clinical condition on admission**									
HH									< 0.001
0		2(0.2)	2(1.7)				0(0)		
1		202(19)	15(13)				187(20)		
2		586(56)	62(52)				524(57)		
3		182(18)	31(26)				151(16)		
4		64(6.2)	9(7.5)				55(6.0)		
5		3(0.3)	1(0.8)				2(0.2)		
Fisher									< 0.001
0		158(15)	2(1.7)				156(17)		
1		143(14)	19(16)				124(13)		
2		376(36)	44(37)				332(36)		
3		214(21)	20(17)				194(21)		
4		148(14)	35(29)				113(12)		
WFNS									0.007
1		771(74)	73(61)				698(76)		
2		106(10)	21(18)				85(9.2)		
3		43(4.1)	8(6.7)				35(3.8)		
4		75(7.2)	12(10)				63(6.9)		
5		44(4.2)	6(5.0)				38(4.1)		
mRS									< 0.001
0		603(58)	37(31)				566(62)		
1		201(19)	45(38)				156(17)		
2		60(5.8)	8(6.7)				52(5.7)		
3		41(3.9)	11(9.2)				30(3.3)		
4		45(4.3)	6(5.0)				39(4.2)		
5		84(8.1)	12(10)				72(7.8)		
6		5(0.5)	1(0.8)				4(0.4)		
GCS		15 [14, 15]	15 [13, 15]				15 [15]		< 0.001
**Clinical routine examination data**									
PT		11.8[11.1, 12.6]	12.4[11.6, 13]				11.7[11.1, 12.5]		< 0.001
APTT		28.3[25.4, 32.5]	30.1[26.7, 33.2]				28.2[25.3, 32.4]		0.032
D-Dimer		2.45[1.09, 44]	289.2[2.53, 682.18]				2.16[1.04,7.47]		< 0.001
WBC		8.37[6.55, 11.03]	9.85[7.75, 12.44]				8.22[6.42, 10.82]		< 0.001
NLR		4.946[3.183, 9.341]	8.788[5.074,15.116]				4.687[3.093,8.523]		< 0.001
PNR		25.930[18.832,36.293]	22.222[15.509,27.393]				26.589[19.203,37.000]		<0.001
**DCI**		239(23.003)	60(50.000)				179(19.478)		< 0.001

### Model performance

**Fig. 2:**
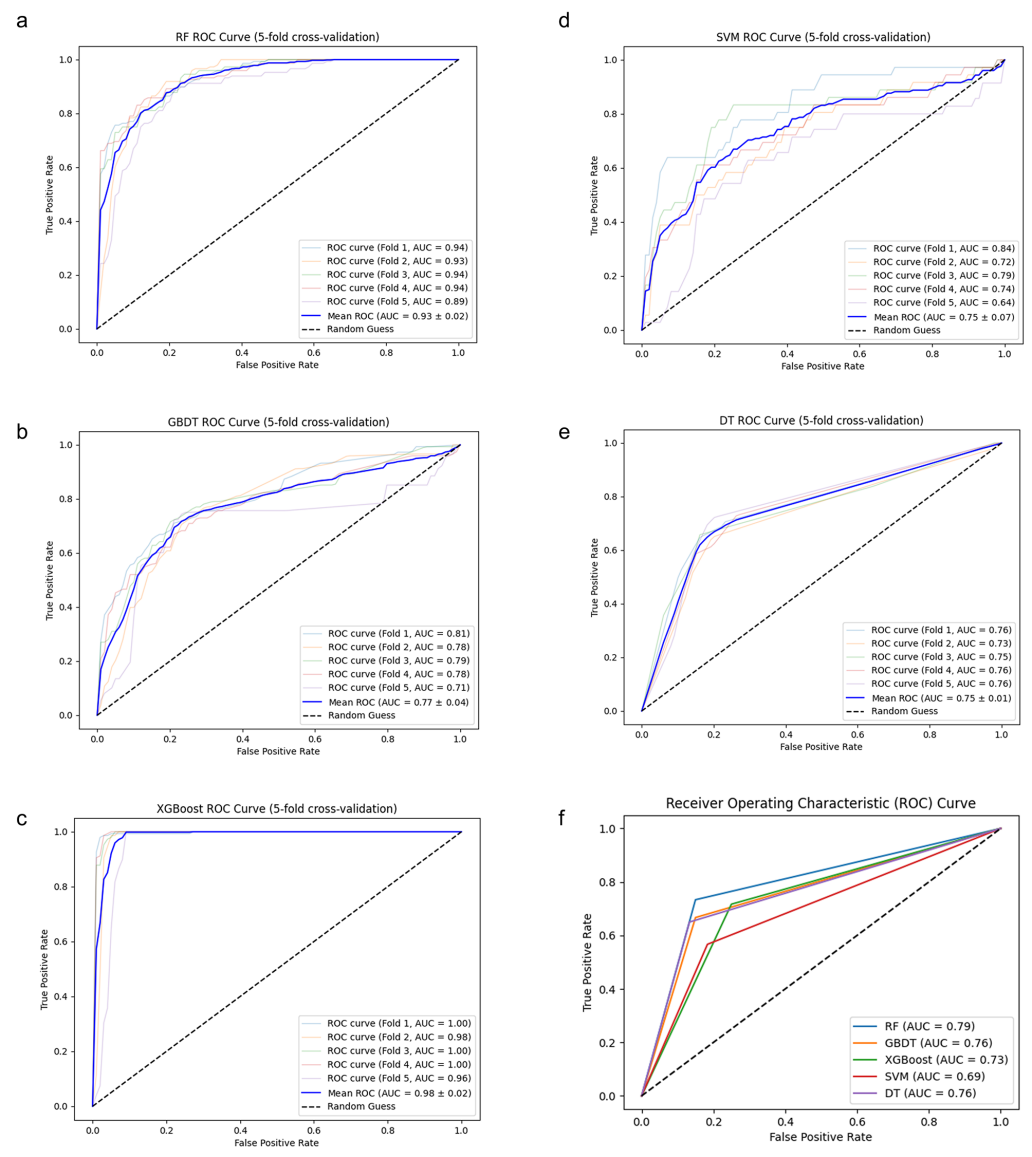
Machine learning model performance in identifying DCI. (**a**-**e**) The AUC of RF, GBDT, XGBoost, SVM, and DT in the training and testing datasets. (**f**) The AUC of ML methods in the test datasets.

The performance of the five models is shown in Fig. 2. Upon running each model against the training cohort, the RF model achieved an ROC(receiver operating characteristic curve) of 0.93, the XGBoost model achieved an ROC of 0.98, the GBDT model achieved an ROC of 0.77, the SVM model achieved an ROC of 0.75. The DT model achieved an ROC of 0.75. All five models exhibit reliable performance.

The five models were validated against the test set. When tested against the patient population of the test set, the AUC was 0.79 for the RF model, 0.73 for the XGBoost model, 0.76 for the GBDT model, 0.69 for the SVM model, and 0.76 for the DT model. The performance of the RF model is as follows: the model’s accuracy is 0.79, the sensitivity is 0.73, the specificity is 0.85, the precision is 0.83, and the F1 score is 0.78. The performance of the XGBoost model is as follows: the model’s accuracy is 0.73, the sensitivity is 0.72, the specificity is 0.75, the precision is 0.74, and the F1 score is 0.73. The performance of the GBDT model is as follows: the model?s accuracy is 0.76, the sensitivity is 0.67, the specificity is 0.85, the precision is 0.82, and the F1 score is 0.73. The performance of the SVM model is as follows: the model?s accuracy is 0.69, the sensitivity is 0.57, the specificity is 0.82, the precision is 0.76, and the F1 score is 0.65. The performance of the DT model is as follows: the model?s accuracy is 0.76, the sensitivity is 0.65, the specificity is 0.87, the precision is 0.83, and the F1 score is 0.73 (Table 2). After comparison, the model with the best performance is RF. 

**Table 2:** Summary of model performances on the test set

	Accuracy	Sensitivity	Specificity	Precision	F1score	AUROC
RF	**0.79**	**0.73**	0.85	**0.83**	**0.78**	**0.79**
XGBoost	0.73	0.72	0.75	0.74	0.73	0.73
GBDT	0.76	0.67	0.85	0.82	0.73	0.76
SVM	0.69	0.57	0.82	0.76	0.65	0.69
DT	0.76	0.65	**0.87**	**0.83**	0.73	0.76

To calculate these performance metrics, patients with DCI are considered true positives, and patients without DCI are considered true negatives. Therefore, patients identified by the model as at risk of DCI should be interpreted as requiring some preventive and interventional measures as recommended by their physicians during hospitalization.

**Fig. 3:**
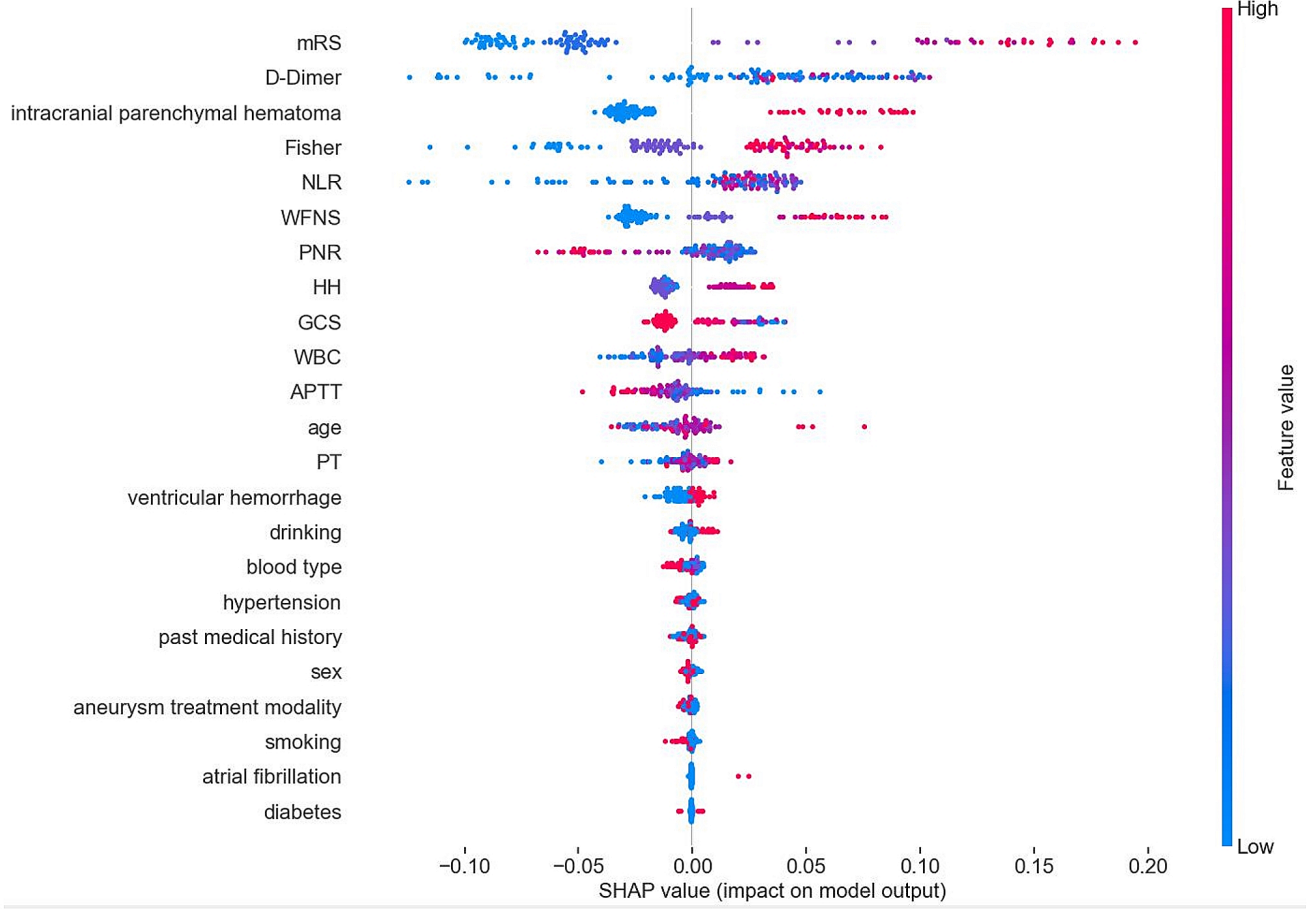
All features of the RF model are shown. Each point on the plot is a Shapley (importance) value for a single patient. The color of each point represents the magnitude and direction of the value of that feature for that patient. The point’s position on the horizontal axis represents the importance and direction of that feature for the prediction for that patient. SHAP summary plot showing the significance of all features for the RF model. The top five most important features are mRS, D-Dimer, intracranial parenchymal hematoma, Fisher score, and NLR

After constructing the prediction models of DCI, we calculated the SHAP values of the RF model with the best performance and used them to determine global feature importance rankings across the study population. Figure.  3 shows all the RF model’s features (determined using Shapley values). We found that the mRS representing the clinical condition on admission was the most crucial feature for the prediction of DCI, with a higher predicted chance occurring when the mRS value was higher. Conversely, for the PNR, the higher the value, the lower the expected probability of DCI. The second important feature was the D-Dimer, with a higher predicted chance when the D-Dimer value was higher. Meanwhile, NLR and Fisher also showed high characteristic importance for DCI prediction, and patients with intracranial parenchymal hematoma showed a higher chance of predicting DCI.

**Table 3:** Collinearity analysis of the RF model’s top 5 most essential variables

variable		VIF
mRS	1.180	
D-Dimer	1.049	
intracranial parenchymal hematoma	1.188	
Fisher	1.273	
NLR	1.083	

To analyze whether the five most important predictive variables, including mRS, D-Dimer, intracranial parenchymal hematoma, NLR, and Fisher score, interacted with each other statistically, we performed a characteristic collinearity analysis (Table 3). The variance inflation factor (VIF) is a measure of the severity of multicollinearity. Generally, greater than 10 means that it has multicollinearity. After analysis, we can see that the top 5 important features do not have multicollinearity, that is, they do not interact with each other statistically.

**Fig. 4:**
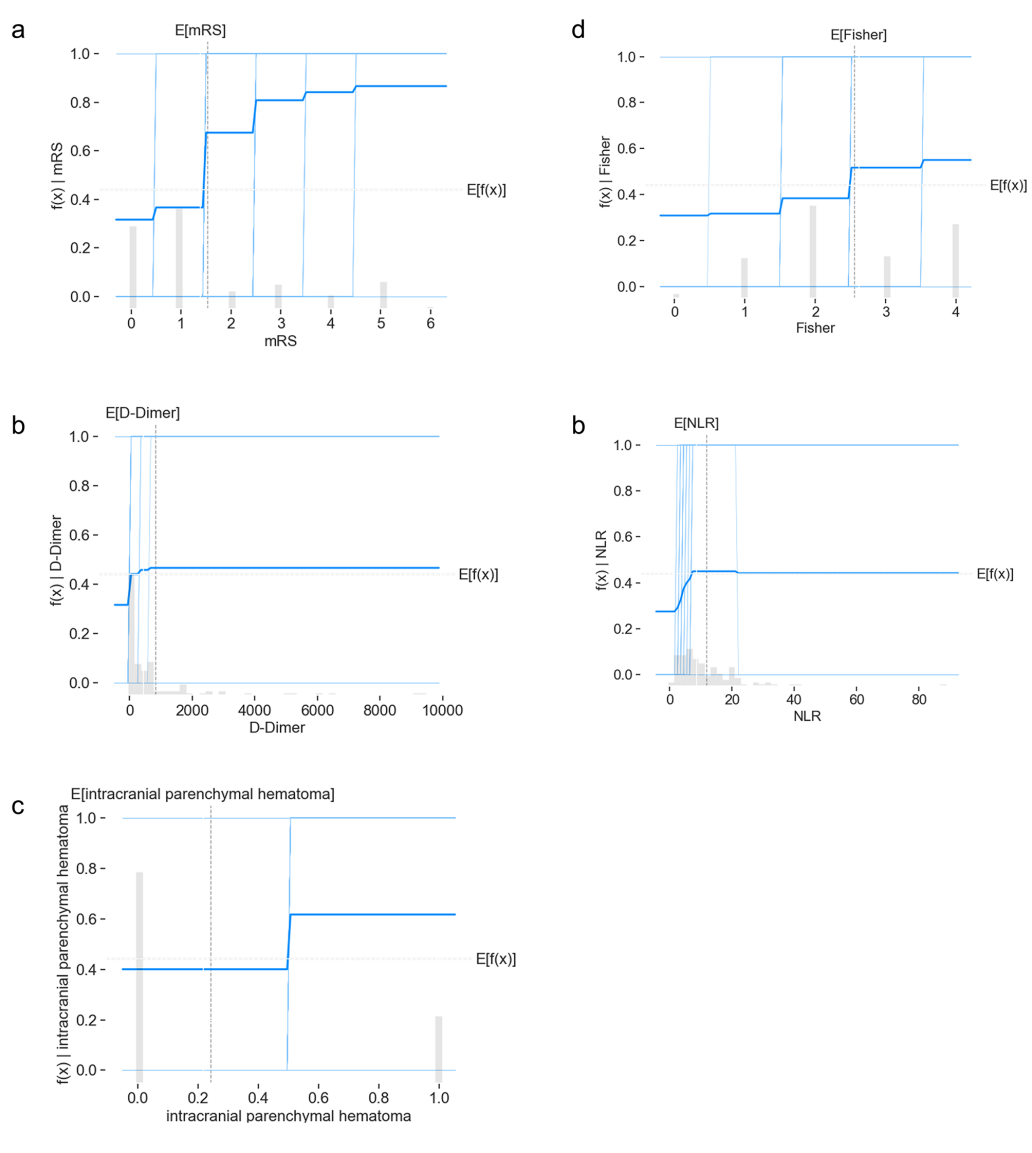
Partial dependence plots of critical features show how a risk factor affects the model prediction. The gray horizontal line in the plot above represents the expected value of the model when applied to the California housing dataset. The vertical gray line represents the average value of the median income feature. The vertical axis is the final prediction of the model. Partial dependence plots for the RF model’s top 5 most essential elements are (**a**) mRS, (**b**) D-Dimer, (**c**) intracranial parenchymal hematoma, (**d**) Fisher, (**e**) NLR

Figure. 4 shows the partial dependence plot of the prediction model’s top 5 most essential features. These results illustrated the model prediction results when the individual feature became large from small. We found that when the mRS value exceeded 2, the probability of DCI was higher (Fig. 4a). As the D-Dimer, Fisher score, and NLR values increased and the PNR value decreased, the model predicted a greater likelihood of DCI, but none of the chances were very high (Fig. 4b, d, e). Patients with intracranial parenchymal hematoma were more likely to develop DCI(Fig. 4c).

## Discussion

To obtain reliable results, we use five ML models. The model with the best performance is selected for feature importance analysis. To prevent any bias that could be introduced by prior assumptions about the features of DCI, we adopted a non- invasive approach to feature selection and minimized manual curation before training and testing our models. As a result of utilizing this methodology, it may not always be apparent why a particular feature is essential to one or more models. However, clinical experts identified several potential themes upon reviewing the elements depicted in Fig. 3.

First, clinical conditions on admission are commonly used to assess the severity of subarachnoid hemorrhage. The mRS is a scale to evaluate neurological recovery and disability after a stroke [14]. It measures the patient’s ability to live independently, including physical function, mobility, and participation in daily life. The mRS score is 0–6, with 0 representing no symptoms and 6 representing death. The higher the score, the worse the patient’s prognosis [15]. Patients with higher mRS scores often exhibit poorer consciousness and limb mobility, which may indicate slower cerebral blood flow and systemic hypercoagulability, potentially leading to a higher risk of DCI [16]. Moreover, a higher Fisher score often represents a more extensive intracerebral, which induces a higher risk of post-SAH vasospasm [17]. Early brain injury due to the hematoma occupancy effect and higher responsiveness of cortical vessels in functional areas to hemorrhagic stimuli may be involved in the development of DCI [18]. Previous studies have demonstrated that DCI is associated with severe vasospasm and more extensive hemorrhage. At the same time, applying vasodilator drugs such as nicardipine, nimodipine, and papaverine can reduce the incidence of DCI [19]. In conclusion, the risk of DCI is higher in poorly clinically graded patients, and the next step of a randomized controlled trial is needed to answer whether prophylactic use of vasodilators could decrease DCI incidence.

Second, post-operation clinical examinations in the top 5 risk factors of the RF model are D-Dimer and NLR. NLR is the ratio of neutrophil count to lymphocyte count in the blood. Neutrophils release inflammatory factors, including reactive oxygen species, cathepsin, matrix metalloproteinase-9, and myeloperoxidase, disrupting the blood-brain barrier, brain edema, and nerve cell damage [20]. NLR represents the balance between innate and adaptive immune responses [21]. NLR > 14.0 (sensitivity 74.5%, specificity 69.3%) was reported to be associated with poor outcomes after aSAH, with better predictive power for DCI when NLR was more significant than 14.3 (sensitivity 87.3%, specificity 48.4%) [10]. Our ML model validates the predictive power of NLR, which had a higher specificity of (sensitivity 73%, specificity 85%) and ranked fifth in predicting DCI. Postoperative monitoring of NLR in SAH patients may be an effective means of reducing DCI, and the efficacy and safety of the metric used as an early warning of intervention need to be confirmed by prospective clinical studies. The dynamic balance between the coagulation and fibrinolytic systems maintains the fluid state of the blood. D-Dimer is a specific degradation product of fibrin monomers cross-linked by activated factor XIII and then hydrolyzed by fibrinolytic enzymes, which, in essence, is a specific marker for the fibrinolytic process [22]. The elevation of D-Dimer is triggered when the body is in a hypercoagulable state or in the presence of thrombosis/hematoma forming, which is more likely to be increased by a more extensive subarachnoid hematoma [18]. Hurth et al. found by ROC curve analysis that D-Dimer>0.445 mg/ml increases the risk of vasospasm and microthrombosis in small vessels, contributing to DCI development among patients in Fisher grade 4. Elevated D-Dimer can be attributed to large intracranial hematomas and reduced physical activity due to impaired consciousness. Therefore, clinical attention needs to be paid to early training to promote awakening and cerebrospinal fluid drainage to reduce D-Dimer. These post-operation clinical examinations are easy to measure and timely monitor, and appropriate interventions can help to reduce the incidence of DCI.

Third, Two features of cerebral hemorrhage were included: intracranial parenchymal hematoma and ventricular hemorrhage, respectively. However, the importance of the two features for the models differs significantly. For the RF model, intracranial parenchymal hematoma is of relatively high priority, ranked third contributor to DCI, while the significance of ventricular hemorrhage is relatively low. It has been shown in several studies that the site of aneurysm rupture in patients who developed DCI was usually located in the anterior cerebral artery/middle cerebral artery [18, 23]. Ruptured aneurysms in these locations tend to form intracranial parenchymal hematomas, consistent with our observations. Ventricular hemorrhage only contacts the intraventricular choroid plexus and does not directly affect cortical functional areas, which may explain the low predictive significance of this marker. Clinically, we need to pay more attention to the risk of DCI in patients with intracranial parenchymal hematoma.

Interestingly, this study’s predictive value of the aneurysm surgery method (clamping or embolization) for DCI was relatively low (the fourth button-down risk factor, Fig. 3**)**. Two meta-analyses reached different conclusions regarding whether clamping increased the incidence of vasospasm [24, 25]. The advantage of clamping is the maximum intraoperative removal of the hematoma and the reduction of blood irritation to the brain tissue. However, the disruption of brain structure by surgery stimulates the release of inflammatory mediators, and manipulating the cerebral artery may aggravate vasospasm [25]. Interventional embolization may disrupt hemodynamics, and endovascular stenting carries a risk of thrombosis, so the impact on DCI remains unclear. In reality, these differences may come from the patient’s status at the time of admission rather than the surgery. Joos et al. found that Patients undergoing clamping procedures have worse WFNS and Fisher scores at the entrance [17]. We are the first to demonstrate that aneurysm treatment modality is not a significant factor in delayed cerebral ischemia through real-world extensive data analysis, and the findings will provide evidence for clinical decision-making.

The AUC value of the best model is 0.79, there is no significant advantage of this value compared to other reported values in previous studies. However, we observed that a series of metrics including accuracy, sensitivity, specificity, precision, and F1 score were all well-balanced. The algorithm demonstrates excellent balance and strong capabilities in correctly classifying and excluding false positives, which holds positive implications for practical applications. It is important to note that a comprehensive evaluation of the model using multiple metrics is crucial, as a single metric might not fully capture the performance characteristics of the model. Therefore, despite not achieving the expected advantage in terms of AUC value, when considering the performance across other metrics, we can still maintain a positive assessment and recognize the potential application value of the model.

Regarding the involved classifiers, RF performed the best in DCI prediction among aSAH patients. It is modeled by randomly selecting features and sample subsets. Compared with other ML algorithms, RF has been reported to have the advantage of dealing with high dimensionality, multiple features, and large data volume and can effectively reduce the overfitting problem [26, 27]. Recent years have witnessed the increasing application of RF in various medical data analytics, such as malignant middle cerebral artery infarction disease prediction [28] and the developing neonatal seizure prediction models [29]. Compared to existing literature, our model achieves a higher sensitivity rate [30] than previous studies. Moreover, our larger sample size enhances the reliability of our findings [31]. 

Our study employs ML models, including RF, to predict DCI post-aSAH, identifying key predictive variables such as mRS, D-Dimer, intracranial parenchymal hematoma, NLR, and Fisher score. Compared to traditional regression analyses, our ML approach offers enhanced capability in capturing complex, non-linear relationships and interactions among these variables. While regression analysis has provided foundational insights into DCI risk factors, our ML model underscores both the confirmation of previously recognized predictors and the identification of nuanced interactions and potential novel predictors not as readily discernible through traditional methods.

Particularly, the RF model demonstrated superior performance with an AUC of 0.79, validating the significant role of the aforementioned variables in DCI risk. This not only aligns with existing literature but also extends our understanding by highlighting the intricate dynamics at play, which may not be fully appreciated through conventional statistical approaches. For example, the model’s ability to account for the cumulative impact of both individual and interactive effects of risk factors offers a more comprehensive risk stratification tool for clinical use.

One of the innovations in our study is that the data were collected from multicenters and included multiple pre- and post-operation indicators, including NLR associated with inflammation and D-Dimer associated with coagulation disorders. The above two indicators are the first to be demonstrated to correlate with DCI through an ML approach. Moreover, we used multiple ML models to compete, and the results indicated that the RF model showed better prediction performance (accuracy 0.79, sensitivity 0.73, specificity 0.85, precision 0.83, F1 score 0.78). Finally, ML based on multicenter real-world data first revealed the aneurysm surgery method has little to do with the DCI occurrence after aSAH in the present study.

### Limitations

As insufficient evidence brought by our retrospective study, it is essential to consider conducting a more extensive prospective study to validate our results. Because DCI appears to occur in a minority of patients with aSAH, our model will always produce a non-trivial number of false positives. As more data are available about patients with DCI over time, we will be better able to characterize false positives and negatives in future iterations. Finally, it is challenging to interpret the ML models. The “black box” issue is a common challenge in various fields [32]. Although we ranked variables using SHAP analysis, comprehending the decision-making procedure of ML is critical to boosting our understanding of the disease process.

## Conclusion

In this multicenter study to predict DCI occurrence, we compared several ML methods, among which RF performed best. In addition, we located the top 5 impact indicators (mRS, D-Dimer, intracranial parenchymal hematoma, Fisher score, and NLR) to identify high risk for DCI after aSAH. Interestingly, ML found that differences in aneurysm treatment modality hardly affected the incidence of DCI. These findings help clinicians predict high-risk patients with DCI after aSAH surgery and redress the abnormal parameters promptly to prevent DCI occurrence.

### Electronic supplementary material

Below is the link to the electronic supplementary material.

Supplementary Material 1

Supplementary Material 2

## Data Availability

No datasets were generated or analysed during the current study.

## Abbreviations

DCI: Delayed cerebral ischemia
aSAH: Aneurysmal subarachnoid hemorrhage
WBCs: White blood cells
ML: Machine Learning
RF: Random Forest
XGBoost: eXtreme Gradient Boosting
SVM: Support Vector Machines
GBDT: Gradient Boosting Decision Tree
DT: Decision Tree
TCD: Transcranial Doppler
HH: Hunt and Hess grade
WFNS: World Federation of Neurosurgical Societies
mRS: Modified Rankin Scale
GCS: Glasgow Coma Scale
PT: Prothrombin time
APTT: Activated partial thromboplastin time
NLR: Neutrophil/lymphocyte ratio
PNR: Platelet/neutrophil ratio
AUC: Area under the receiver operating characteristic curve
SHAP: Shapley additive explanation
