# Supplementary material for: Predicting who has delayed cerebral ischemia after aneurysmal subarachnoid hemorrhage using machine learning approach: a multicenter, retrospective cohort study
Source: BMC Neurol. 2024 May 27;24:177. doi: 10.1186/s12883-024-03630-2 (PMC11129362; doi:10.1186/s12883-024-03630-2)
Supplement: Supplementary file 1 — Supplementary Material 1 [file 12883_2024_3630_MOESM1_ESM.docx]

**Supplemental Table 1** Sensitivity analysis assesses the importance of input variables

| variable | ST | ST_conf | |
| --- | --- | --- | --- |
| **mRS** | 0.667 | 0.062 | |
| **Fisher** | 0.294 | 0.045 | |
| age | 0.253 | 0.036 | |
| APTT | 0.189 | 0.037 | |
| **D-Dimer** | 0.124 | 0.027 | |
| aneurysm treatment modality | 0.124 | 0.034 | |
|  |  |  | |
| PNR | 0.116 | | 0.028 |
| GCS | 0.109 | | 0.026 |
| PT | 0.105 | | 0.025 |
| diabetes | 0.097 | | 0.027 |
| **intracranial parenchymal hematoma** | 0.091 | | 0.02 |
| **NLR** | 0.081 | | 0.022 |
| HH | 0.077 | | 0.022 |
| blood type | 0.075 | | 0.023 |
| WBC | 0.073 | | 0.024 |
| WFNS | 0.061 | | 0.021 |
| sex | 0.061 | | 0.021 |
| smoking | 0.053 | | 0.018 |
| hypertension | 0.051 | | 0.018 |
| drinking | 0.045 | | 0.016 |
| ventricular hemorrhage | 0.037 | | 0.017 |
| past medical history | 0.028 | | 0.013 |
| atrial fibrillation | 0.004 | | 0.005 |
