# Supplementary material for: Predicting who has delayed cerebral ischemia after aneurysmal subarachnoid hemorrhage using machine learning approach: a multicenter, retrospective cohort study
Source: BMC Neurol. 2024 May 27;24:177. doi: 10.1186/s12883-024-03630-2 (PMC11129362; doi:10.1186/s12883-024-03630-2)
Supplement: Supplementary file 2 — Supplementary Material 2 [file 12883_2024_3630_MOESM2_ESM.docx]

**Supplemental Table 2** raw data of HH and Fisher

| **HH** | **Fisher** | **DCI** |
| --- | --- | --- |
| 2 | 1 | No |
| 2 | 1 | No |
| 3 | 2 | No |
| 2 | 3 | No |
| 2 | 2 | No |
| 2 | 1 | No |
| 2 | 1 | No |
| 1 | 1 | No |
| 2 | 1 | No |
| 3 | 3 | No |
| 3 | 2 | No |
| 2 | 2 | No |
| 2 | 4 | No |
| 2 | 2 | No |
| 3 | 2 | No |
| 1 | 1 | No |
| 2 | 1 | No |
| 2 | 2 | No |
| 3 | 4 | No |
| 4 | 4 | No |
| 2 | 2 | No |
| 2 | 3 | No |
| 2 | 1 | No |
| 2 | 2 | No |
| 2 | 2 | No |
| 2 | 2 | No |
| 1 | 2 | No |
| 2 | 4 | No |
| 1 | 2 | No |
| 2 | 2 | No |
| 2 | 2 | No |
| 2 | 3 | No |
| 2 | 1 | No |
| 2 | 2 | No |
| 4 | 1 | No |
| 2 | 1 | No |
| 2 | 4 | No |
| 2 | 2 | No |
| 2 | 2 | No |
| 2 | 3 | No |
| 2 | 3 | No |
| 2 | 0 | No |
| 1 | 1 | No |
| 1 | 0 | No |
| 2 | 3 | No |
| 1 | 1 | No |
| 2 | 0 | No |
| 2 | 4 | No |
| 1 | 2 | No |
| 2 | 1 | No |
| 2 | 0 | No |
| 2 | 4 | No |
| 2 | 1 | No |
| 2 | 3 | No |
| 1 | 1 | No |
| 1 | 2 | No |
| 2 | 2 | No |
| 2 | 3 | No |
| 1 | 2 | No |
| 2 | 2 | No |
| 4 | 1 | No |
| 2 | 2 | No |
| 2 | 3 | No |
| 2 | 4 | No |
| 2 | 1 | No |
| 2 | 2 | No |
| 2 | 1 | No |
| 2 | 4 | No |
| 3 | 2 | No |
| 4 | 3 | No |
| 1 | 0 | No |
| 2 | 2 | No |
| 2 | 3 | No |
| 1 | 1 | No |
| 2 | 3 | No |
| 1 | 0 | No |
| 2 | 2 | No |
| 1 | 0 | No |
| 2 | 1 | No |
| 1 | 1 | No |
| 2 | 3 | No |
| 1 | 0 | No |
| 2 | 2 | No |
| 2 | 4 | No |
| 2 | 2 | No |
| 2 | 2 | No |
| 2 | 3 | No |
| 2 | 2 | No |
| 4 | 3 | No |
| 1 | 0 | No |
| 2 | 1 | No |
| 1 | 2 | No |
| 1 | 2 | No |
| 2 | 0 | No |
| 2 | 2 | No |
| 2 | 3 | No |
| 2 | 1 | No |
| 2 | 1 | No |
| 1 | 0 | No |
| 1 | 1 | No |
| 1 | 0 | No |
| 3 | 2 | No |
| 2 | 3 | No |
| 2 | 3 | No |
| 4 | 3 | No |
| 2 | 0 | No |
| 2 | 1 | No |
| 2 | 2 | No |
| 2 | 1 | No |
| 3 | 4 | No |
| 4 | 4 | Yes |
| 2 | 2 | No |
| 2 | 0 | No |
| 2 | 1 | No |
| 2 | 2 | No |
| 2 | 2 | No |
| 4 | 4 | No |
| 3 | 4 | Yes |
| 4 | 3 | Yes |
| 2 | 3 | No |
| 2 | 0 | No |
| 2 | 2 | No |
| 1 | 0 | No |
| 2 | 2 | No |
| 1 | 0 | No |
| 2 | 3 | No |
| 2 | 0 | No |
| 1 | 2 | No |
| 2 | 2 | No |
| 3 | 3 | No |
| 3 | 3 | Yes |
| 2 | 2 | No |
| 4 | 4 | Yes |
| 3 | 4 | No |
| 1 | 2 | No |
| 4 | 4 | Yes |
| 2 | 1 | No |
| 3 | 3 | No |
| 2 | 0 | No |
| 3 | 3 | Yes |
| 2 | 2 | No |
| 3 | 2 | Yes |
| 2 | 3 | No |
| 3 | 2 | Yes |
| 2 | 3 | No |
| 2 | 0 | No |
| 2 | 2 | No |
| 2 | 3 | No |
| 2 | 2 | No |
| 1 | 2 | Yes |
| 2 | 1 | No |
| 1 | 2 | No |
| 1 | 2 | No |
| 2 | 0 | No |
| 3 | 4 | Yes |
| 2 | 2 | No |
| 2 | 2 | No |
| 1 | 4 | No |
| 2 | 0 | No |
| 1 | 1 | No |
| 1 | 1 | No |
| 3 | 1 | No |
| 2 | 1 | No |
| 2 | 0 | No |
| 1 | 2 | No |
| 2 | 3 | No |
| 3 | 2 | Yes |
| 1 | 2 | No |
| 2 | 3 | No |
| 3 | 2 | No |
| 3 | 4 | No |
| 3 | 3 | No |
| 2 | 2 | No |
| 2 | 2 | No |
| 2 | 0 | No |
| 2 | 2 | No |
| 2 | 2 | No |
| 2 | 3 | No |
| 2 | 4 | No |
| 4 | 4 | Yes |
| 2 | 1 | No |
| 1 | 3 | Yes |
| 3 | 3 | No |
| 3 | 4 | Yes |
| 3 | 2 | No |
| 3 | 4 | Yes |
| 2 | 2 | No |
| 1 | 0 | No |
| 2 | 3 | No |
| 1 | 3 | Yes |
| 3 | 2 | Yes |
| 2 | 0 | No |
| 2 | 0 | No |
| 2 | 0 | No |
| 4 | 4 | Yes |
| 2 | 2 | No |
| 2 | 1 | No |
| 1 | 0 | No |
| 2 | 0 | No |
| 3 | 4 | No |
| 2 | 2 | No |
| 2 | 1 | No |
| 4 | 4 | No |
| 1 | 1 | No |
| 2 | 3 | No |
| 2 | 0 | No |
| 4 | 3 | Yes |
| 1 | 1 | No |
| 2 | 2 | No |
| 1 | 2 | No |
| 1 | 2 | No |
| 1 | 2 | No |
| 4 | 4 | Yes |
| 2 | 4 | Yes |
| 4 | 4 | Yes |
| 2 | 2 | No |
| 3 | 2 | No |
| 3 | 1 | No |
| 2 | 2 | No |
| 2 | 1 | No |
| 2 | 2 | Yes |
| 3 | 2 | No |
| 2 | 1 | No |
| 2 | 2 | No |
| 2 | 0 | No |
| 2 | 2 | No |
| 2 | 1 | No |
| 1 | 2 | No |
| 1 | 0 | No |
| 3 | 1 | No |
| 2 | 0 | No |
| 1 | 0 | No |
| 1 | 0 | No |
| 2 | 1 | No |
| 2 | 2 | No |
| 2 | 2 | No |
| 1 | 2 | No |
| 2 | 2 | No |
| 2 | 1 | No |
| 2 | 1 | Yes |
| 2 | 0 | No |
| 3 | 2 | No |
| 2 | 2 | Yes |
| 2 | 0 | No |
| 2 | 4 | No |
| 1 | 0 | No |
| 3 | 1 | No |
| 2 | 3 | Yes |
| 2 | 2 | Yes |
| 1 | 2 | Yes |
| 2 | 0 | No |
| 2 | 2 | No |
| 2 | 2 | No |
| 1 | 0 | No |
| 1 | 1 | Yes |
| 2 | 2 | No |
| 2 | 2 | No |
| 1 | 1 | No |
| 2 | 2 | No |
| 1 | 0 | No |
| 4 | 3 | No |
| 1 | 2 | No |
| 2 | 4 | No |
| 2 | 3 | No |
| 3 | 3 | No |
| 4 | 2 | Yes |
| 2 | 2 | No |
| 1 | 2 | No |
| 2 | 4 | No |
| 2 | 1 | No |
| 2 | 2 | No |
| 1 | 2 | No |
| 1 | 2 | No |
| 2 | 0 | No |
| 2 | 0 | No |
| 2 | 1 | No |
| 3 | 2 | No |
| 2 | 3 | Yes |
| 2 | 4 | No |
| 2 | 1 | No |
| 2 | 1 | No |
| 2 | 3 | No |
| 2 | 3 | No |
| 2 | 2 | No |
| 3 | 3 | No |
| 1 | 0 | No |
| 1 | 2 | No |
| 2 | 3 | No |
| 3 | 2 | No |
| 4 | 1 | Yes |
| 1 | 0 | No |
| 3 | 4 | No |
| 4 | 3 | No |
| 2 | 3 | No |
| 3 | 4 | No |
| 2 | 1 | No |
| 2 | 2 | No |
| 2 | 0 | No |
| 2 | 0 | No |
| 1 | 0 | No |
| 1 | 0 | No |
| 4 | 3 | Yes |
| 2 | 2 | No |
| 2 | 1 | Yes |
| 1 | 0 | No |
| 2 | 2 | No |
| 2 | 2 | No |
| 2 | 1 | No |
| 3 | 1 | Yes |
| 2 | 0 | Yes |
| 3 | 2 | No |
| 3 | 3 | No |
| 2 | 4 | No |
| 2 | 0 | No |
| 2 | 3 | Yes |
| 3 | 0 | No |
| 2 | 3 | No |
| 3 | 2 | Yes |
| 1 | 0 | No |
| 2 | 2 | Yes |
| 2 | 3 | No |
| 2 | 2 | Yes |
| 1 | 2 | No |
| 3 | 3 | Yes |
| 4 | 4 | Yes |
| 2 | 4 | Yes |
| 1 | 2 | Yes |
| 3 | 4 | Yes |
| 2 | 1 | No |
| 1 | 3 | No |
| 1 | 2 | Yes |
| 2 | 1 | No |
| 2 | 4 | No |
| 2 | 1 | No |
| 1 | 1 | No |
| 1 | 2 | No |
| 2 | 0 | No |
| 3 | 2 | Yes |
| 3 | 4 | No |
| 2 | 2 | No |
| 3 | 4 | Yes |
| 2 | 2 | No |
| 2 | 2 | No |
| 2 | 3 | No |
| 4 | 4 | Yes |
| 2 | 2 | No |
| 1 | 2 | No |
| 4 | 4 | Yes |
| 2 | 1 | No |
| 2 | 2 | No |
| 1 | 2 | No |
| 1 | 1 | Yes |
| 2 | 2 | No |
| 1 | 0 | Yes |
| 3 | 3 | Yes |
| 2 | 1 | No |
| 2 | 2 | Yes |
| 1 | 0 | No |
| 1 | 1 | Yes |
| 2 | 3 | No |
| 4 | 4 | Yes |
| 3 | 0 | No |
| 3 | 3 | No |
| 2 | 2 | No |
| 2 | 3 | No |
| 1 | 1 | No |
| 2 | 1 | No |
| 1 | 3 | No |
| 2 | 0 | No |
| 1 | 1 | No |
| 2 | 0 | No |
| 1 | 0 | No |
| 2 | 2 | No |
| 2 | 2 | Yes |
| 3 | 4 | Yes |
| 2 | 1 | No |
| 1 | 2 | No |
| 1 | 0 | No |
| 2 | 4 | Yes |
| 4 | 4 | No |
| 2 | 2 | No |
| 3 | 3 | Yes |
| 2 | 0 | No |
| 2 | 2 | Yes |
| 2 | 0 | No |
| 2 | 4 | No |
| 1 | 1 | No |
| 2 | 3 | No |
| 1 | 3 | No |
| 1 | 4 | No |
| 2 | 0 | No |
| 4 | 4 | Yes |
| 3 | 2 | No |
| 4 | 2 | No |
| 1 | 0 | No |
| 3 | 3 | Yes |
| 1 | 2 | No |
| 2 | 3 | No |
| 3 | 4 | Yes |
| 2 | 0 | Yes |
| 2 | 2 | No |
| 2 | 2 | Yes |
| 4 | 2 | No |
| 2 | 1 | No |
| 2 | 2 | Yes |
| 4 | 3 | No |
| 1 | 0 | No |
| 3 | 1 | No |
| 1 | 2 | No |
| 2 | 0 | No |
| 4 | 3 | Yes |
| 2 | 0 | Yes |
| 1 | 0 | No |
| 2 | 3 | No |
| 2 | 0 | No |
| 4 | 3 | Yes |
| 1 | 3 | No |
| 1 | 0 | No |
| 2 | 2 | Yes |
| 2 | 3 | No |
| 1 | 2 | No |
| 2 | 2 | No |
| 2 | 2 | No |
| 2 | 3 | No |
| 4 | 4 | Yes |
| 2 | 3 | No |
| 2 | 0 | No |
| 2 | 2 | Yes |
| 2 | 2 | No |
| 2 | 2 | No |
| 2 | 0 | No |
| 2 | 2 | No |
| 2 | 0 | No |
| 2 | 3 | No |
| 1 | 2 | No |
| 1 | 1 | No |
| 2 | 1 | No |
| 1 | 0 | No |
| 1 | 0 | No |
| 2 | 1 | No |
| 2 | 2 | Yes |
| 2 | 3 | No |
| 2 | 0 | No |
| 2 | 3 | No |
| 2 | 4 | Yes |
| 1 | 0 | No |
| 2 | 3 | Yes |
| 1 | 0 | No |
| 1 | 3 | No |
| 1 | 3 | No |
| 1 | 2 | Yes |
| 3 | 2 | No |
| 1 | 4 | Yes |
| 2 | 4 | Yes |
| 2 | 0 | No |
| 2 | 2 | No |
| 2 | 2 | No |
| 3 | 3 | No |
| 3 | 2 | No |
| 1 | 2 | Yes |
| 1 | 0 | No |
| 2 | 2 | No |
| 2 | 2 | No |
| 1 | 2 | No |
| 2 | 0 | No |
| 4 | 3 | No |
| 1 | 1 | No |
| 3 | 3 | Yes |
| 1 | 2 | No |
| 2 | 2 | No |
| 1 | 3 | Yes |
| 2 | 2 | No |
| 2 | 0 | No |
| 1 | 1 | No |
| 3 | 1 | No |
| 1 | 0 | No |
| 2 | 2 | No |
| 2 | 4 | No |
| 1 | 2 | No |
| 3 | 3 | No |
| 2 | 1 | No |
| 1 | 0 | No |
| 3 | 3 | Yes |
| 5 | 4 | No |
| 2 | 2 | No |
| 2 | 3 | No |
| 1 | 2 | No |
| 2 | 2 | Yes |
| 1 | 3 | Yes |
| 2 | 4 | No |
| 2 | 2 | No |
| 1 | 0 | No |
| 2 | 1 | No |
| 2 | 4 | No |
| 2 | 0 | No |
| 3 | 2 | No |
| 1 | 2 | No |
| 2 | 0 | No |
| 2 | 1 | No |
| 2 | 2 | Yes |
| 1 | 2 | No |
| 4 | 3 | Yes |
| 2 | 0 | No |
| 2 | 3 | Yes |
| 2 | 2 | No |
| 1 | 0 | No |
| 2 | 2 | No |
| 2 | 0 | No |
| 3 | 4 | Yes |
| 2 | 1 | No |
| 1 | 2 | No |
| 2 | 1 | No |
| 2 | 3 | Yes |
| 2 | 3 | Yes |
| 3 | 3 | No |
| 3 | 4 | Yes |
| 2 | 4 | No |
| 2 | 0 | No |
| 1 | 3 | No |
| 1 | 2 | No |
| 2 | 3 | No |
| 3 | 3 | No |
| 2 | 0 | No |
| 2 | 1 | No |
| 3 | 3 | No |
| 2 | 3 | No |
| 3 | 4 | No |
| 2 | 1 | No |
| 1 | 3 | No |
| 1 | 0 | No |
| 3 | 3 | Yes |
| 2 | 3 | No |
| 1 | 0 | No |
| 2 | 4 | No |
| 2 | 0 | No |
| 2 | 0 | No |
| 2 | 3 | Yes |
| 2 | 1 | No |
| 2 | 2 | No |
| 2 | 0 | No |
| 2 | 3 | No |
| 2 | 1 | No |
| 2 | 2 | No |
| 3 | 2 | No |
| 2 | 3 | Yes |
| 4 | 3 | No |
| 2 | 2 | Yes |
| 2 | 2 | No |
| 2 | 2 | No |
| 2 | 0 | No |
| 1 | 0 | No |
| 2 | 3 | Yes |
| 2 | 0 | Yes |
| 1 | 0 | No |
| 1 | 0 | No |
| 4 | 3 | No |
| 2 | 1 | No |
| 1 | 0 | No |
| 2 | 4 | Yes |
| 2 | 4 | Yes |
| 2 | 2 | No |
| 2 | 3 | No |
| 2 | 1 | No |
| 1 | 0 | No |
| 1 | 0 | No |
| 1 | 0 | No |
| 4 | 4 | Yes |
| 2 | 0 | No |
| 2 | 3 | No |
| 3 | 4 | No |
| 2 | 1 | No |
| 1 | 4 | No |
| 1 | 4 | No |
| 2 | 0 | No |
| 2 | 1 | No |
| 2 | 3 | No |
| 2 | 0 | No |
| 1 | 2 | No |
| 3 | 4 | No |
| 2 | 3 | No |
| 2 | 0 | No |
| 2 | 3 | Yes |
| 2 | 1 | No |
| 1 | 2 | No |
| 2 | 0 | No |
| 2 | 3 | No |
| 2 | 0 | Yes |
| 1 | 0 | No |
| 2 | 0 | No |
| 2 | 3 | Yes |
| 1 | 2 | No |
| 4 | 2 | Yes |
| 2 | 2 | No |
| 2 | 3 | Yes |
| 2 | 2 | No |
| 3 | 2 | No |
| 3 | 4 | No |
| 2 | 0 | No |
| 3 | 4 | No |
| 1 | 2 | Yes |
| 2 | 2 | No |
| 2 | 2 | Yes |
| 3 | 3 | Yes |
| 2 | 2 | No |
| 3 | 3 | No |
| 2 | 2 | Yes |
| 2 | 2 | No |
| 2 | 0 | No |
| 1 | 0 | No |
| 2 | 3 | No |
| 1 | 3 | No |
| 2 | 0 | No |
| 2 | 3 | No |
| 2 | 2 | No |
| 3 | 2 | No |
| 2 | 1 | No |
| 2 | 2 | No |
| 1 | 2 | Yes |
| 2 | 3 | No |
| 1 | 2 | No |
| 2 | 2 | No |
| 3 | 3 | No |
| 2 | 0 | No |
| 1 | 3 | No |
| 2 | 1 | No |
| 3 | 4 | No |
| 2 | 0 | No |
| 2 | 3 | No |
| 1 | 2 | Yes |
| 1 | 0 | No |
| 1 | 2 | No |
| 1 | 2 | No |
| 2 | 0 | No |
| 2 | 2 | No |
| 2 | 1 | No |
| 1 | 2 | No |
| 3 | 3 | No |
| 2 | 0 | No |
| 1 | 2 | No |
| 4 | 2 | No |
| 1 | 3 | No |
| 1 | 0 | No |
| 2 | 0 | No |
| 2 | 2 | No |
| 2 | 4 | No |
| 3 | 3 | No |
| 2 | 2 | No |
| 2 | 2 | No |
| 2 | 0 | No |
| 4 | 2 | Yes |
| 1 | 1 | Yes |
| 2 | 2 | No |
| 4 | 3 | Yes |
| 2 | 3 | No |
| 2 | 0 | No |
| 2 | 0 | No |
| 1 | 1 | Yes |
| 2 | 0 | No |
| 2 | 3 | Yes |
| 2 | 2 | Yes |
| 1 | 1 | Yes |
| 3 | 4 | No |
| 1 | 1 | No |
| 2 | 2 | No |
| 1 | 0 | No |
| 5 | 3 | Yes |
| 3 | 0 | No |
| 2 | 1 | No |
| 2 | 1 | No |
| 2 | 1 | No |
| 2 | 0 | No |
| 2 | 1 | No |
| 2 | 3 | No |
| 2 | 3 | No |
| 2 | 0 | No |
| 2 | 1 | No |
| 2 | 2 | No |
| 2 | 2 | No |
| 4 | 3 | Yes |
| 3 | 3 | Yes |
| 3 | 4 | Yes |
| 2 | 0 | Yes |
| 3 | 4 | No |
| 2 | 2 | No |
| 2 | 0 | No |
| 4 | 4 | Yes |
| 2 | 3 | Yes |
| 3 | 3 | Yes |
| 2 | 2 | No |
| 2 | 2 | Yes |
| 2 | 0 | No |
| 2 | 2 | No |
| 2 | 4 | No |
| 2 | 4 | Yes |
| 2 | 0 | No |
| 2 | 3 | Yes |
| 2 | 0 | No |
| 2 | 3 | No |
| 2 | 3 | Yes |
| 2 | 2 | No |
| 2 | 2 | No |
| 1 | 0 | No |
| 1 | 0 | No |
| 2 | 2 | No |
| 2 | 2 | No |
| 1 | 1 | No |
| 1 | 1 | No |
| 2 | 1 | No |
| 1 | 0 | No |
| 2 | 0 | No |
| 2 | 0 | No |
| 3 | 2 | No |
| 1 | 2 | No |
| 2 | 2 | No |
| 2 | 3 | No |
| 2 | 0 | No |
| 2 | 2 | No |
| 2 | 0 | No |
| 2 | 2 | No |
| 2 | 2 | No |
| 2 | 2 | No |
| 1 | 1 | No |
| 2 | 2 | No |
| 2 | 2 | No |
| 2 | 2 | No |
| 3 | 3 | No |
| 1 | 0 | No |
| 1 | 2 | No |
| 2 | 2 | No |
| 2 | 0 | No |
| 1 | 1 | No |
| 1 | 2 | No |
| 2 | 4 | No |
| 2 | 2 | No |
| 2 | 4 | No |
| 3 | 3 | No |
| 2 | 2 | No |
| 2 | 3 | No |
| 1 | 2 | No |
| 2 | 1 | No |
| 4 | 4 | Yes |
| 2 | 2 | Yes |
| 2 | 0 | Yes |
| 2 | 3 | Yes |
| 3 | 3 | Yes |
| 2 | 3 | Yes |
| 2 | 3 | Yes |
| 2 | 4 | Yes |
| 3 | 0 | Yes |
| 4 | 4 | Yes |
| 3 | 2 | Yes |
| 3 | 2 | Yes |
| 2 | 3 | Yes |
| 4 | 4 | Yes |
| 3 | 4 | Yes |
| 2 | 3 | Yes |
| 2 | 3 | Yes |
| 2 | 2 | Yes |
| 2 | 2 | Yes |
| 1 | 3 | Yes |
| 2 | 3 | Yes |
| 2 | 1 | Yes |
| 3 | 3 | Yes |
| 3 | 3 | Yes |
| 4 | 3 | Yes |
| 1 | 3 | Yes |
| 1 | 4 | Yes |
| 2 | 4 | Yes |
| 1 | 3 | Yes |
| 2 | 4 | Yes |
| 1 | 2 | Yes |
| 2 | 0 | Yes |
| 3 | 4 | Yes |
| 2 | 3 | Yes |
| 2 | 2 | Yes |
| 2 | 4 | Yes |
| 1 | 3 | Yes |
| 2 | 0 | Yes |
| 1 | 2 | Yes |
| 3 | 2 | Yes |
| 2 | 2 | No |
| 2 | 2 | No |
| 3 | 3 | No |
| 3 | 3 | No |
| 1 | 2 | No |
| 3 | 3 | No |
| 2 | 2 | No |
| 2 | 2 | No |
| 2 | 2 | No |
| 3 | 3 | No |
| 3 | 2 | No |
| 2 | 2 | No |
| 3 | 4 | No |
| 2 | 2 | No |
| 3 | 2 | No |
| 3 | 2 | No |
| 3 | 2 | No |
| 2 | 2 | No |
| 2 | 4 | No |
| 3 | 3 | No |
| 2 | 2 | No |
| 2 | 1 | No |
| 3 | 2 | No |
| 2 | 2 | No |
| 3 | 4 | No |
| 2 | 2 | No |
| 2 | 3 | No |
| 2 | 2 | No |
| 3 | 3 | No |
| 3 | 3 | No |
| 2 | 3 | No |
| 3 | 3 | No |
| 2 | 2 | No |
| 3 | 3 | No |
| 2 | 2 | No |
| 3 | 3 | No |
| 3 | 2 | No |
| 2 | 3 | No |
| 2 | 3 | No |
| 3 | 2 | No |
| 2 | 2 | No |
| 3 | 4 | No |
| 4 | 4 | No |
| 3 | 2 | No |
| 3 | 3 | No |
| 3 | 4 | No |
| 3 | 4 | No |
| 2 | 4 | No |
| 2 | 2 | No |
| 2 | 2 | No |
| 3 | 3 | No |
| 2 | 2 | No |
| 3 | 2 | No |
| 3 | 3 | No |
| 3 | 2 | No |
| 3 | 2 | No |
| 3 | 3 | No |
| 2 | 3 | No |
| 2 | 2 | No |
| 3 | 2 | No |
| 2 | 2 | No |
| 3 | 3 | No |
| 2 | 3 | No |
| 2 | 1 | No |
| 2 | 4 | No |
| 2 | 3 | No |
| 2 | 2 | No |
| 4 | 3 | Yes |
| 3 | 3 | No |
| 2 | 2 | No |
| 2 | 2 | Yes |
| 3 | 2 | No |
| 2 | 2 | Yes |
| 4 | 3 | No |
| 3 | 4 | No |
| 2 | 2 | No |
| 2 | 2 | No |
| 2 | 2 | No |
| 2 | 2 | No |
| 3 | 2 | No |
| 3 | 4 | Yes |
| 2 | 2 | No |
| 2 | 2 | No |
| 1 | 1 | No |
| 3 | 3 | No |
| 4 | 4 | No |
| 3 | 4 | No |
| 2 | 2 | No |
| 4 | 4 | No |
| 2 | 2 | No |
| 3 | 3 | No |
| 2 | 2 | No |
| 2 | 2 | No |
| 2 | 2 | No |
| 2 | 3 | No |
| 2 | 2 | Yes |
| 2 | 2 | No |
| 2 | 2 | No |
| 2 | 2 | No |
| 2 | 2 | No |
| 2 | 2 | Yes |
| 2 | 2 | No |
| 3 | 4 | No |
| 2 | 2 | No |
| 2 | 2 | No |
| 2 | 2 | No |
| 2 | 2 | No |
| 2 | 2 | No |
| 2 | 3 | No |
| 2 | 1 | No |
| 2 | 2 | No |
| 2 | 2 | No |
| 3 | 3 | No |
| 2 | 0 | No |
| 1 | 2 | No |
| 1 | 2 | No |
| 1 | 1 | No |
| 1 | 1 | No |
| 2 | 2 | No |
| 2 | 2 | No |
| 1 | 1 | No |
| 2 | 2 | No |
| 2 | 1 | No |
| 3 | 4 | No |
| 2 | 2 | No |
| 1 | 2 | No |
| 3 | 2 | No |
| 3 | 2 | No |
| 2 | 1 | No |
| 2 | 4 | No |
| 2 | 2 | No |
| 2 | 2 | No |
| 2 | 2 | No |
| 1 | 2 | No |
| 2 | 2 | No |
| 2 | 2 | No |
| 2 | 2 | No |
| 3 | 3 | No |
| 4 | 4 | Yes |
| 2 | 2 | No |
| 2 | 1 | No |
| 2 | 2 | No |
| 2 | 1 | No |
| 1 | 2 | No |
| 2 | 2 | No |
| 3 | 1 | No |
| 2 | 2 | No |
| 1 | 1 | No |
| 3 | 4 | No |
| 2 | 1 | No |
| 2 | 4 | No |
| 2 | 2 | No |
| 2 | 1 | No |
| 1 | 0 | No |
| 3 | 4 | No |
| 2 | 1 | No |
| 2 | 1 | No |
| 2 | 1 | No |
| 2 | 2 | No |
| 4 | 4 | Yes |
| 4 | 4 | Yes |
| 2 | 2 | Yes |
| 3 | 2 | Yes |
| 1 | 1 | Yes |
| 2 | 4 | Yes |
| 3 | 3 | Yes |
| 3 | 2 | Yes |
| 2 | 4 | Yes |
| 4 | 4 | Yes |
| 2 | 3 | Yes |
| 3 | 4 | Yes |
| 1 | 0 | Yes |
| 2 | 4 | Yes |
| 2 | 2 | Yes |
| 2 | 3 | Yes |
| 2 | 4 | Yes |
| 3 | 3 | Yes |
| 2 | 2 | Yes |
| 2 | 3 | Yes |
| 1 | 2 | No |
| 1 | 1 | No |
| 3 | 3 | No |
| 0 | 1 | No |
| 2 | 2 | No |
| 3 | 2 | No |
| 3 | 2 | No |
| 2 | 1 | No |
| 2 | 1 | No |
| 2 | 3 | No |
| 4 | 3 | No |
| 2 | 1 | No |
| 3 | 3 | No |
| 2 | 1 | No |
| 2 | 2 | No |
| 2 | 1 | No |
| 0 | 1 | No |
| 2 | 2 | No |
| 2 | 3 | No |
| 2 | 2 | No |
| 4 | 4 | Yes |
| 3 | 4 | Yes |
| 2 | 3 | Yes |
| 3 | 4 | Yes |
| 3 | 4 | Yes |
| 3 | 4 | Yes |
| 2 | 3 | Yes |
| 2 | 3 | Yes |
| 3 | 3 | Yes |
| 3 | 4 | Yes |
| 2 | 4 | Yes |
| 3 | 4 | Yes |
| 4 | 4 | Yes |
| 4 | 3 | Yes |
| 3 | 4 | Yes |
| 3 | 2 | Yes |
| 2 | 4 | Yes |
| 3 | 4 | Yes |
| 5 | 4 | Yes |
| 4 | 4 | Yes |
| 2 | 2 | No |
| 1 | 2 | No |
| 2 | 2 | No |
| 3 | 4 | No |
| 2 | 2 | No |
| 2 | 2 | No |
| 2 | 2 | No |
| 2 | 1 | No |
| 3 | 3 | No |
| 2 | 2 | No |
| 2 | 2 | No |
| 2 | 2 | No |
| 1 | 2 | No |
| 4 | 4 | No |
| 2 | 2 | No |
| 2 | 2 | No |
| 2 | 2 | No |
| 3 | 4 | No |
| 1 | 2 | No |
| 3 | 4 | No |
| 3 | 3 | Yes |
| 3 | 4 | Yes |
| 3 | 4 | Yes |
| 1 | 2 | Yes |
| 3 | 4 | Yes |
| 3 | 4 | Yes |
| 1 | 3 | Yes |
| 1 | 2 | Yes |
| 1 | 2 | Yes |
| 1 | 2 | Yes |
| 2 | 2 | Yes |
| 2 | 2 | Yes |
| 2 | 2 | Yes |
| 2 | 2 | Yes |
| 2 | 4 | Yes |
| 2 | 2 | Yes |
| 2 | 4 | Yes |
| 2 | 3 | Yes |
| 2 | 3 | Yes |
| 2 | 2 | Yes |
